# Field Testing of Biohybrid Robotic Jellyfish to Demonstrate Enhanced Swimming Speeds

**DOI:** 10.3390/biomimetics5040064

**Published:** 2020-11-21

**Authors:** Nicole W. Xu, James P. Townsend, John H. Costello, Sean P. Colin, Brad J. Gemmell, John O. Dabiri

**Affiliations:** 1 Department of Bioengineering, School of Engineering and School of Medicine, Stanford University, Stanford, CA 94305, USA; 2 Graduate Aerospace Laboratories (GALCIT), California Institute of Technology, Pasadena, CA 91125, USA; jodabiri@caltech.edu; 3 Whitman Center, Marine Biological Laboratory, Woods Hole, MA 02543, USA; jptownsendii@gmail.com (J.P.T.); costello@providence.edu (J.H.C.); scolin@rwu.edu (S.P.C.); 4 Department of Biology, Providence College, Providence, RI 02918, USA; 5 Department of Marine Biology and Environmental Science, Roger Williams University, Bristol, RI 02809, USA; 6 Department of Integrative Biology, University of South Florida, Tampa, FL 33620, USA; bgemmell@usf.edu; 7 Department of Mechanical Engineering, California Institute of Technology, Pasadena, CA 91125, USA

**Keywords:** jellyfish, biohybrid robot, swimming speed, ocean monitoring

## Abstract

Biohybrid robotic designs incorporating live animals and self-contained microelectronic systems can leverage the animals’ own metabolism to reduce power constraints and act as natural chassis and actuators with damage tolerance. Previous work established that biohybrid robotic jellyfish can exhibit enhanced speeds up to 2.8 times their baseline behavior in laboratory environments. However, it remains unknown if the results could be applied in natural, dynamic ocean environments and what factors can contribute to large animal variability. Deploying this system in the coastal waters of Massachusetts, we validate and extend prior laboratory work by demonstrating increases in jellyfish swimming speeds up to 2.3 times greater than their baseline, with absolute swimming speeds up to 6.6 ± 0.3 cm s^−1^. These experimental swimming speeds are predicted using a hydrodynamic model with morphological and time-dependent input parameters obtained from field experiment videos. The theoretical model can provide a basis to choose specific jellyfish with desirable traits to maximize enhancements from robotic manipulation. With future work to increase maneuverability and incorporate sensors, biohybrid robotic jellyfish can potentially be used to track environmental changes in applications for ocean monitoring.

## 1. Introduction

With ocean acidification altering animal behavior and function [1,2] and temperature-induced biodiversity changes in marine environments [3,4], new tools can expand efforts to track markers of climate change in more sensitive or previously unexplored areas of the ocean [5]. Traditional ocean monitoring tools, such as autonomous underwater vehicles (AUVs) and remotely operated vehicles (ROVs), offer invaluable opportunities to explore the ocean. For example, prior work using AUVs has yielded observations of deep-sea animal communities over multiple decades [6], and ROVs have been used to monitor anthropogenic disturbances of ecosystems [7] and capture gelatinous midwater animals with soft robotic arms [8]. Despite advantages such as speed and reliability [9,10], AUVs and ROVs are still limited in confined spaces and fragile environments, such as near coral reefs or in caves, where debris can cause severe damage to the vehicles [8,11]. These technologies can also cost thousands of dollars and require specialized operational personnel [12].

In conjunction with AUVs and ROVs, other tools that offer alternative strategies can be developed to expand human capabilities to monitor a variety of ocean environments. One potential solution is to take inspiration from biological organisms, which offer advantages in energy efficiency, maneuverability, and stealth compared to extant robotic systems [13,14]. Bioinspired soft robots can potentially address issues in power consumption [13,15] and leave wakes that mimic the wakes of marine life, with potential to minimally perturb surrounding wildlife. Examples of bioinspired aquatic robots include robotic fish [16,17,18], manta rays [19,20,21], sea stars [22,23], and jellyfish [24,25,26,27,28,29], including systems that have been deployed in real-world environments [16,24].

In particular, moon jellyfish (*Aurelia aurita*) are a compelling model organism for building robots because of the limited energy required for locomotion. *A. aurita* is a species of moon jellyfish that comprise a flexible oblate bell, composed of mesoglea (gelatinous structural tissue that primarily comprises water and extracellular proteins) with a singular muscle layer oriented circumferentially on the subumbrellar surface. The animal has eight natural swim pacemakers located on the bell margin, each of which can independently activate to excite the swim muscle. This initiates the power stroke, in which the muscle contracts to decrease the subumbrellar volume and generate thrust to travel forward. The muscle then rests during the relaxation stroke, returning the jellyfish bell back to its relaxed shape [30]. Induced flow from stopping vortices during a relaxed phase of the swimming cycle provides additional thrust at no increased metabolic input. This process, known as passive energy recapture, allows jellyfish to have the lowest cost of transport (COT), defined as the mass-specific energy input per distance traveled, compared to other animals [31]. 

However, bioinspired robotic constructs mimicking jellyfish still exhibit higher energy costs than their biological analogs [24,26]. An alternative approach is to incorporate live animals into a biohybrid robotic construct, which can then use an inexpensive and simpler microelectronic system to power electrodes that excite an existing biological system, instead of the energy costs and design considerations for using mechanical actuators and chassis. Biological components can also improve damage tolerance using natural tissue regeneration. Instead of relying on the animals’ natural pacemaker system to activate muscle contractions, a robotic system with electrodes that generate square pulse waves of 3.7 V was previously described to incite jellyfish muscle contractions [32]. Prior work has shown that even without arresting endogenous pulses in the animals, driving the jellyfish at various frequencies with the portable swim controller resulted in increased swimming speeds [32].

Although biohybrid designs incorporating live animals are limited by biological constraints, this system can also improve upon biological performance. For example, previous work has demonstrated that by driving jellyfish at faster frequencies than they would normally exhibit themselves, biohybrid robotic jellyfish can increase swimming speeds up to 2.8 times, at only a 10 mW input to the robotic system and twofold increase in metabolic cost to the animal. Biohybrid robotic jellyfish also use less external power per mass than other reported swimming robots in literature [32]. The ubiquity of jellyfish found at various depths, including thousands of meters below surface level [33], offers opportunities to incorporate biohybrid robots to explore new areas of the ocean in the future. This would require only a hardened microelectronic system, as opposed to an entire robot that could be easily damaged in real conditions.

However, previous demonstrations of biohybrid robotic jellyfish were limited to controlled laboratory experiments. Open questions include how natural environmental conditions, such as current and turbulence, affect swimming performance relative to laboratory results in quiescent conditions, and the feasibility of future ocean monitoring using this integrated swim controller and live animal design. We conducted a series of vertical swimming experiments in the coastal waters of Massachusetts to test the effect of externally driven swim controller frequencies on vertical swimming speeds in the ocean. We hypothesized that even in the presence of surface currents, increasing swimming frequency would increase swimming speeds up until a limit, in which altered swim kinematics would result in decreased swimming speeds. A hydrodynamic model was also developed to demonstrate predictive capabilities.

## 2. Materials and Methods

### 2.1. Animal Care

*A. aurita*, originally housed in facilities at Stanford University (animal husbandry details described in [32]), were shipped overnight to the Marine Biological Laboratory (MBL) in Woods Hole, MA. Animals were subsequently stored at room temperature, 21 °C, in standard 5 gal plastic buckets filled with filtered natural seawater from the Atlantic Ocean and fed naupliar brine shrimp for one hour before daily water changes. 

### 2.2. Biohybrid Robotic System

**Figure 1:**
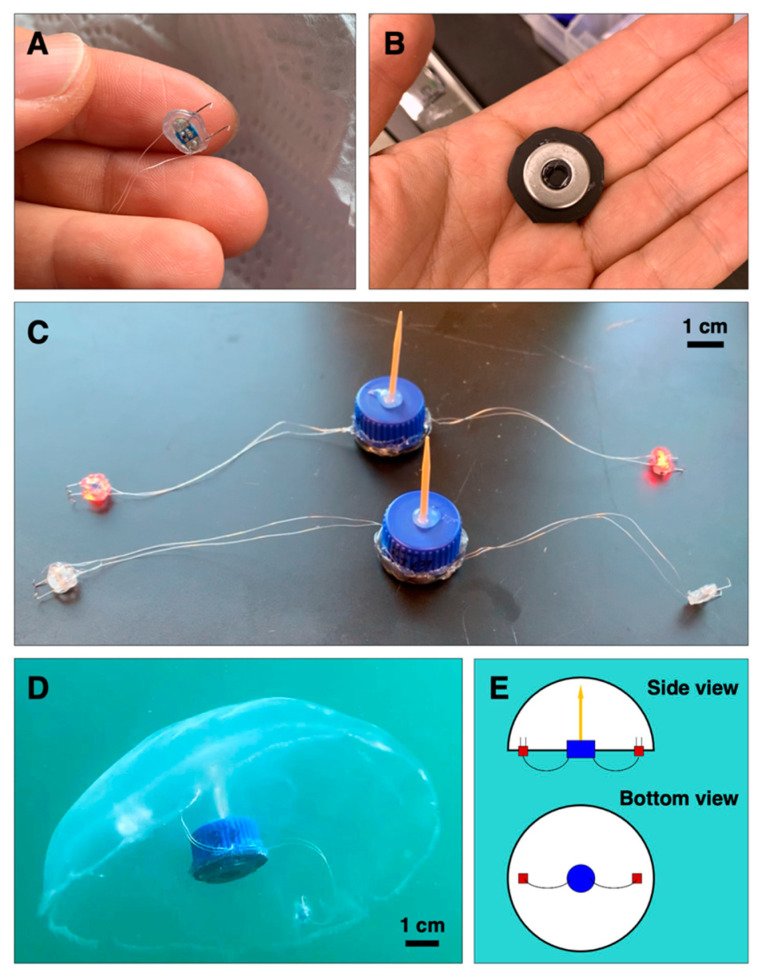
Robotic system using microelectronics. The biohybrid robotic system, adapted from [32], with two new features: (**A**) hooked electrode wire tips instead of straight tips in the previous design, and (**B**) a weighted polypropylene cap to change the ballast and improve the robustness of the system under field conditions, as opposed to no flow in laboratory tank experiments. (**C**) Two of the fully integrated robotic systems with new modifications are shown. The electrodes shown in the back are active (red LEDs, or light-emitting diodes, are on). (**D**) Representative biohybrid robotic jellyfish, comprising the live animal and microelectronic system with two electrodes. (**E**) Side and bottom view schematics of the biohybrid robotic jellyfish. The animal is illustrated in white, swim controller housing in blue, electrodes in red, and wooden pin in yellow.

We adapted the robotic system described in [32] for use in field experiments. The swim controller comprised a mini processor (TinyLily, TinyCircuits, Akron, OH, USA) and 10 mAh lithium polymer cell (GM201212, PowerStream Technology Inc., Orem, UT, USA) in plastic housing made entirely from polypropylene pieces (Figure 1B) sealed with hot melt adhesives, as opposed to the previous design with Parafilm M. The housing was ballasted with stainless steel washers to keep the system neutrally buoyant in seawater. Two electrodes were assembled using perfluoroalkoxy-coated silver wires and platinum rod tips (A-M Systems, Sequim, WA, USA) connected in series to red light-emitting diodes (LEDs); (TinyLily 0402, TinyCircuits, Akron, OH, USA) as a visualization tool. Platinum wire tips were hooked to improve attachment (Figure 1A), an additional design feature to secure the swim controller to the animal in field conditions. Examples of the swim controllers are shown in Figure 1C. 

The robotic system was attached to the jellyfish bell in three locations: a wooden pin connected to the housing was inserted into the center of the manubrium from the subumbrellar surface, and each electrode was inserted into the subumbrellar tissue.

### 2.3. Field Experiments

**Figure 2:**
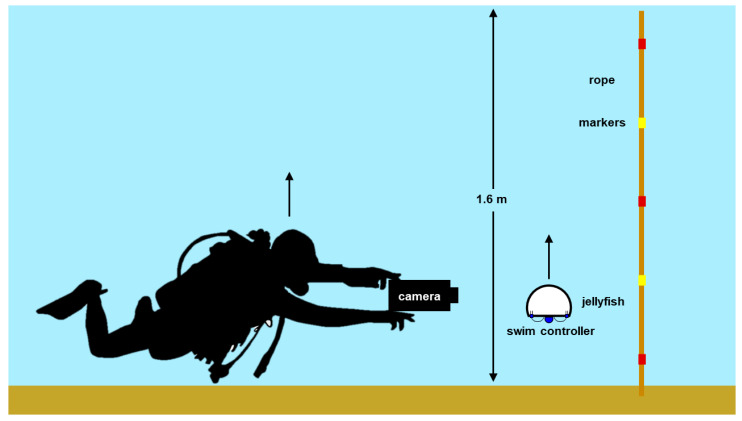
Setup of field experiments. Simplified schematic of the experimental setup, including a scientific diver holding a camera that tracks a biohybrid robotic jellyfish (swim controller and jellyfish) swimming upwards from the ocean floor to the surface, 1.6 m in depth. A rope with alternating red and yellow markers is used to track displacement during image analysis.

Preliminary field tests were conducted in water <1 m in depth to determine the appropriate ballast of the system and robustness of waterproofing techniques on active microelectronics. Subsequent field experiments were conducted in 1.6 m depth water in Woods Hole, MA (dive coordinates 41°31′29.1″ N latitude, 70°40′23.8″ W longitude) and involved a minimum of two scientific scuba divers and one person on shore. One diver maneuvered the animals (*n* = 2) into the starting position (initially at the ocean bottom) near a rope with alternating red and yellow markers at every 30.5 cm, as a known scale for image analysis. Another diver operated a camera system to track the animal and the background rope markers as the biohybrid robotic jellyfish swam upwards to the ocean surface. Videos were recorded in 1920 × 1080 resolution at 30 fps using a Sony AX100 (Sony, Tokyo, Japan) in a Gates AX100 Underwater Housing (Gates Underwater Housing, Poway, CA, USA) on the first dive. An additional *n =* 2 animals were recorded on a second dive for further observations of animal behavior, and were recorded on an iPhone XS (Apple, Cupertino, CA, USA) in a Kraken Universal Smart Housing (Kraken Sports, London, ON, Canada). A simplified schematic of the experimental setup is illustrated in Figure 2. Animals were monitored to ensure recovery after experiments (for more information, see “Ethical considerations” in Appendix B).

Control cases (0 Hz) for each external swim controller frequency (0.50, 0.75, and 1.00 Hz) were tested by cutting both electrode wires, while keeping the electrodes embedded into the animals to maintain neutral buoyancy. In addition to swim controller frequencies (0, 0.50, 0.75, and 1.00 Hz), measured frequencies of the biohybrid robotic jellyfish were determined by counting the number of animals’ pulses within a given time frame. Swim controller frequencies for each animal and wind conditions for Falmouth, MA [34], are listed in Table 1, and video date are available in the repository listed in Appendix C.

### 2.4. Data Analysis

**Figure 3:**
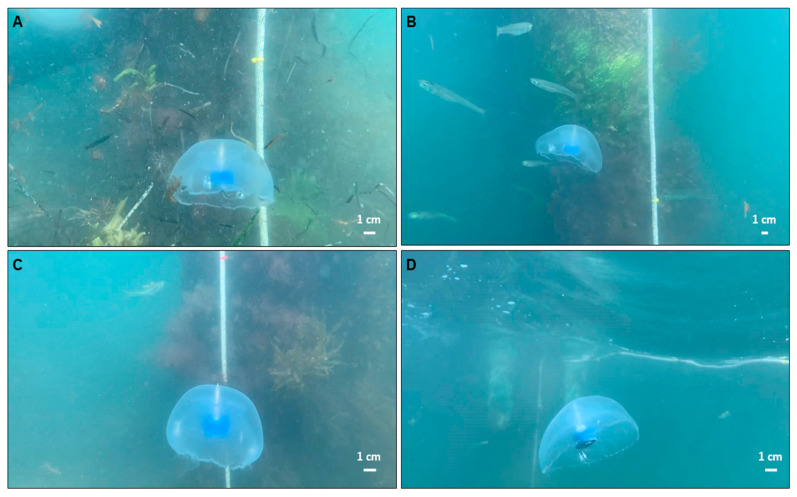
Representative images of biohybrid robotic jellyfish during field experiments. Examples of one biohybrid robotic jellyfish (animal 1) (**A**) initiated at the ocean bed and stimulated at 0.50 Hz, (**B**) swimming toward the ocean surface at 0.50 Hz, (**C**) initiated at the ocean bed and stimulated at 0.50 Hz (second trial), and (**D**) swimming toward the ocean surface at 0.50 Hz (second trial).

**Figure 4:**
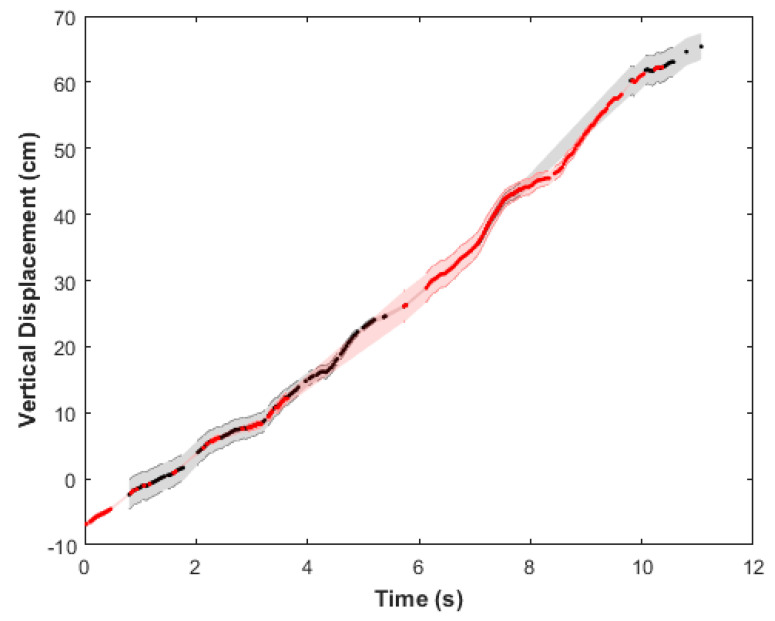
Representative plot tracking animal displacements over time to calculate vertical swimming speeds. Vertical displacement over time of one example jellyfish (animal 1 driven at 0.75 Hz) with respect to the rope markers, with the error propagated from conversions in pixel space to centimeter space. Tracks were assembled by stitching vertical positions using both red and yellow rope markers (shown in red and black, respectively, for improved visualization), to show accuracy in overlap.

**Figure A1:**
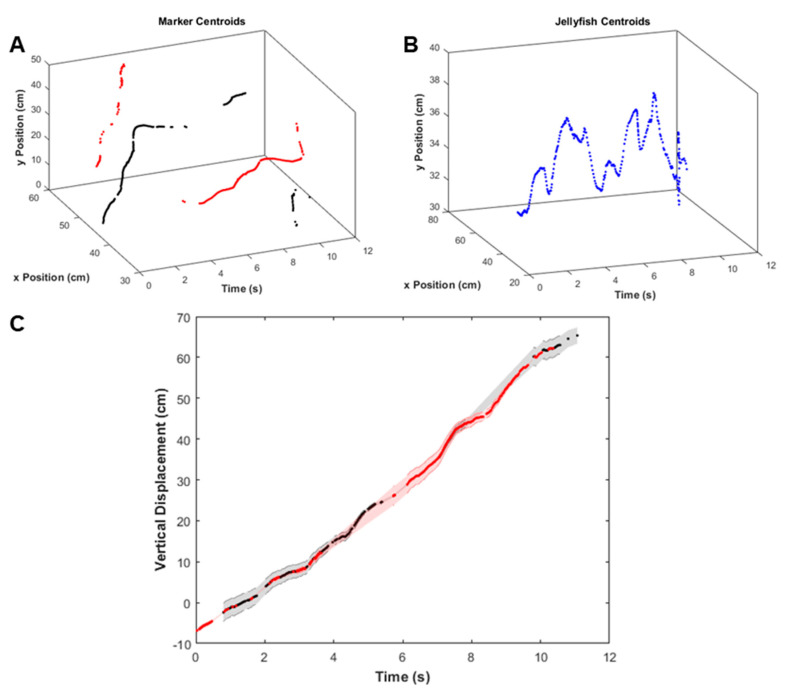
Representative plots tracking animal displacements over time to calculate vertical swimming speeds. To calculate the animals’ displacement over time with respect to the rope, a prominent background feature as a ground truth, we first tracked (**A**) centroids of the red and yellow markers on the rope in each image frame. A sample from one video (animal 1 driven at 0.75 Hz) is shown, with red markers plotted in red and yellow markers plotted in black for improved visualization. (**B**) Centroids of the jellyfish (by tracking the blue polypropylene housing) in each image frame over time. (**C**) Vertical displacement over time of the jellyfish with respect to the rope (data also presented in Figure 4 of the main), with the error propagated from conversions in pixel space to centimeter space. Tracks were assembled by stitching vertical positions using both red and yellow markers (shown in red and black, respectively), to show accuracy in overlap.

Representative images from the videos collected are shown in Figure 3 at various depths during the vertical swimming of each animal. For *n* = 2 animals on the first dive, we tracked centroids of the red and yellow rope markers (see Appendix A, Figure A1A) and housing of the swim controller system (see Appendix A, Figure A1B), assuming pixel-level accuracy in centroids. Vertical displacements of the biohybrid robotic jellyfish over time (Figure 4) were calculated by determining the position of the biohybrid robotic jellyfish with respect to the rope markers. Vertical speeds were calculated using the vertical positions between subsequent time steps and averaged to obtain mean vertical speeds at each test condition. Using vertical speeds, enhancement values were calculated as the measured swimming speed at each experimental condition normalized by the baseline swimming speed. The baseline is defined as the swimming speed of the individual biohybrid robotic jellyfish at 0 Hz, the control case. 

**Figure 5:**
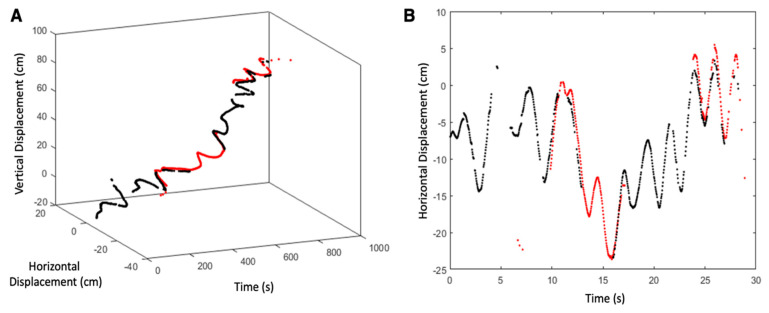
Representative plots tracking animal displacements over time to show horizontal displacements caused by ocean currents. (**A**) An example 2D displacement over time and (**B**) the horizontal component over time from one video (animal 1, driven at 0.50 Hz) to show oscillatory effects from primarily horizontal surface currents.

Similarly, 2D displacement over time was calculated using both vertical and horizontal components (as shown in Figure 5). Although *n* = 4 animals in total were used over two days, rope markers were not visible in videos of *n* = 2 animals on the second day. However, videos from the second dive provide observational data regarding kinematics and confirm swimming speed estimates.

### 2.5. Hydrodynamic Model

As described in [32], a hydrodynamic model was adapted from [35,36] to calculate the velocity (*u*) from a momentum balance using thrust (*T*), drag (*D*), acceleration reaction (*AR*), and inertial forces at a Reynolds number of 325:

$$T-D-AR=m_{j}\frac{du}{dt}$$

in which

$$\;T=\left(\frac{\rho_{w}}{A_{sub}}\right){\left(\frac{dV_{sub}}{dt}\right)}^{2},$$

$$D=\frac{1}{2}C_{d}\rho_{w}A_{j}u^{2},$$

$$AR=\alpha \rho_{j}V_{j}\frac{du}{dt}\;\mathrm{and}\;\alpha ={\left(\frac{2h_{t}}{d_{t}}\right)}^{1.4}$$

with the following terms:

*m_j_* mass of the jellyfish;

*⍴_w_* density of saltwater *=* 1.024 g/cm^3^ at 35 ppt and 21 °C;

*A_sub_* area of the jellyfish subumbrella;

*V_sub_* volume of the jellyfish subumbrella;

*C_d_* drag coefficient *=* 0.42;

*A_j_* area of the jellyfish;

*h_t_* height of the jellyfish;

*d_t_* diameter of the jellyfish;

*⍴_j_* density of the jellyfish;

*V_j_* volume of the jellyfish.

Model inputs included both morphological and time-dependent parameters: relaxed bell height (*h_r_*) and diameter (*d_r_*), maximum change in height between contraction and relaxation states (Δ*h*), maximum change in diameter between relaxation and contraction states (Δ*d*), manubrium tissue height (*h_j_*), contraction time (*t_c_*) defined as the transition from a relaxed to a contracted state, and relaxation time (*t_r_*) defined as the transition from a contracted to a relaxed state.

Velocities from the mechanistic model were calculated for *n* = 2 jellyfish with geometric inputs estimated from experimental videos from the highest measured swimming speeds using ImageJ (National Institutes of Health and the Laboratory for Optical and Computational Instrumentation) and MATLAB (Mathworks). Inputs for the model are listed in Table 2. Mean speeds were calculated from velocities at each time step (30 time steps per second, as a fair comparison to 30 fps in experimental data) for 10 periods. The code is available in the repository listed in Appendix C.

## 3. Results

### 3.1. Externally Driven Jellyfish Can Double Swimming Speeds In Situ

**Figure 6:**
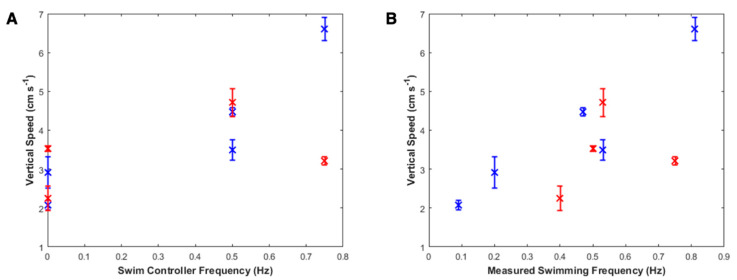
Vertical swimming speeds. (**A**) Vertical swimming speeds over swim controller frequency, the externally driven frequency set by the robotic system. Each animal is represented by a different color (blue or red). Two control measurements were taken for each animal (at 0 Hz), and two videos were recorded at 0.50 Hz for animal 1 (blue). (**B**) Vertical swimming speeds over measured swimming frequency, the summation of both the externally driven frequency set by the robotic system and the animals’ own native pulses. Each animal is represented by a different color (blue or red). Variations in the animals’ baseline frequency can determine limits for robotic manipulation.

**Figure A2:**
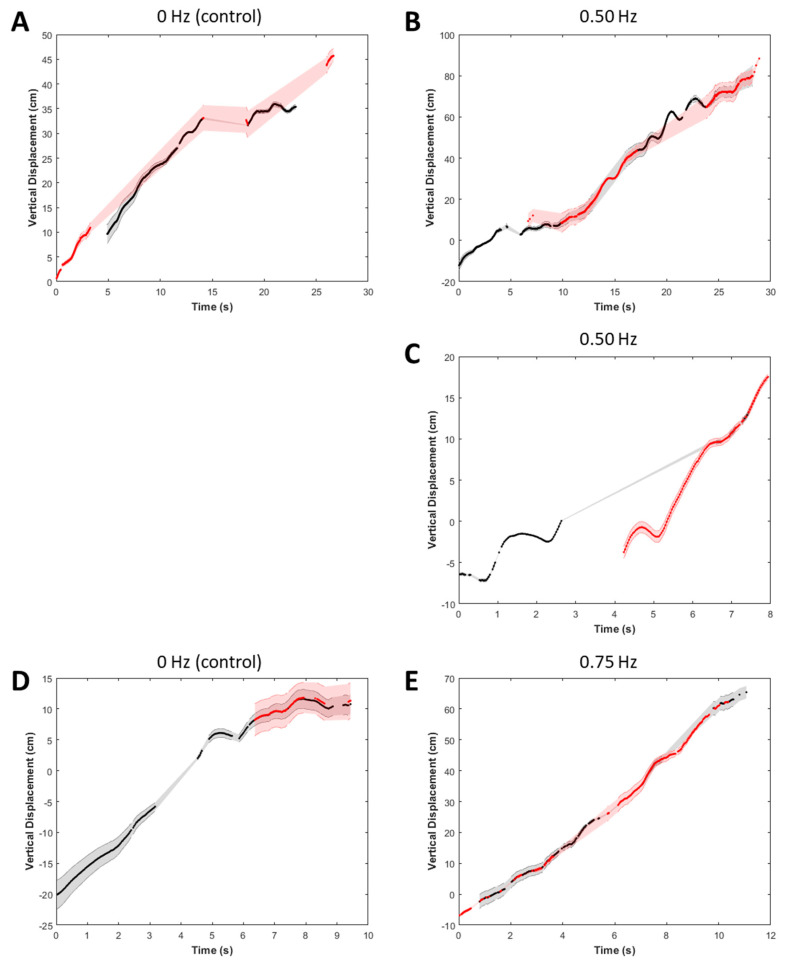
Displacements over time for jellyfish, animal 1, for the swim controller frequencies (**A**) and (**D**) 0 Hz, (**B**) and (**C**) 0.50 Hz, and (**E**) 0.75 Hz. Vertical displacement over time of the jellyfish with respect to the rope, with the error propagated from conversions in pixel space to centimeter space, assuming pixel-level accuracy in centroids. Tracks were assembled by stitching vertical positions using both red and yellow markers (shown in red and black, respectively), to show accuracy in overlap.

**Figure A3:**
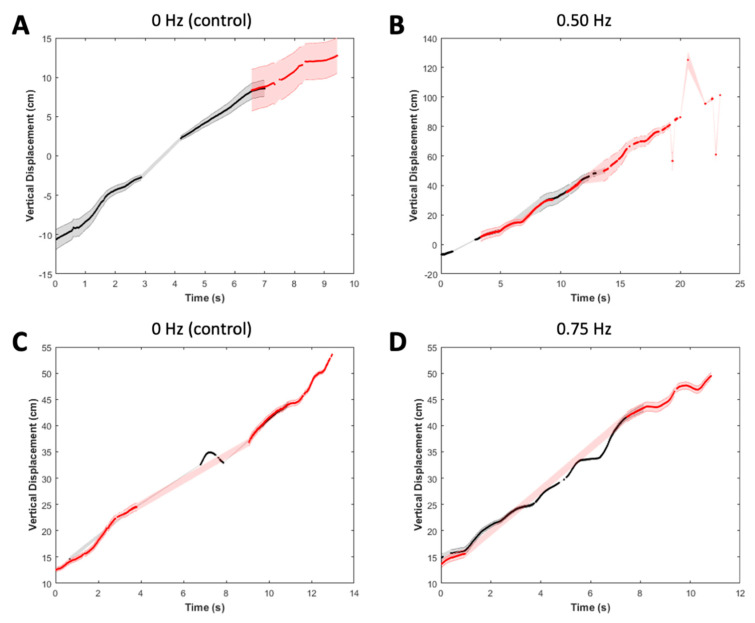
Displacements over time for jellyfish, animal 2, for the swim controller frequencies (**A**) and (**C**) 0 Hz, (**B**) 0.50 Hz, and (**D**) 0.75 Hz. Vertical displacement over time of the jellyfish with respect to the rope, with the error propagated from conversions in pixel space to centimeter space, assuming pixel-level accuracy in centroids. Tracks were assembled by stitching vertical positions using both red and yellow markers (shown in red and black, respectively), to show accuracy in overlap.

From plots of the vertical displacement over time (see Figure 4 for a representative plot, with additional plots available in Figure A2 and Figure A3 in Appendix A), we calculated vertical swimming speeds for three swim controller frequencies: 0 (control with inactive electrodes), 0.50, and 0.75 Hz for *n* = 2 animals, plotted in blue and red, as shown in Figure 6A. Because native animal pulses were not arrested using physical ablation or chemicals to reduce the As biological pacemaker activity, Figure 6B shows the same vertical swimming speeds plotted over the measured swimming frequency (which illustrates the summative effect of the externally driven swim controller frequency and native animal pulses). The maximum vertical swimming speed obtained was 6.6 ± 0.3 cm s^−1^, externally driven at 0.75 Hz, compared to the minimum speed of 2.1 ± 0.1 cm s^−1^ in the absence of external frequency stimulation. Both the maximum and minimum speeds were observed in the same animal (animal 1, labeled in blue), which had a bell diameter of 11.3 ± 1.4 cm with a fineness ratio (defined as the ratio of the bell height to the bell diameter) of 0.39. See Table A1 in Appendix A for more information on the experimental parameters and tabular results.

**Figure 7:**
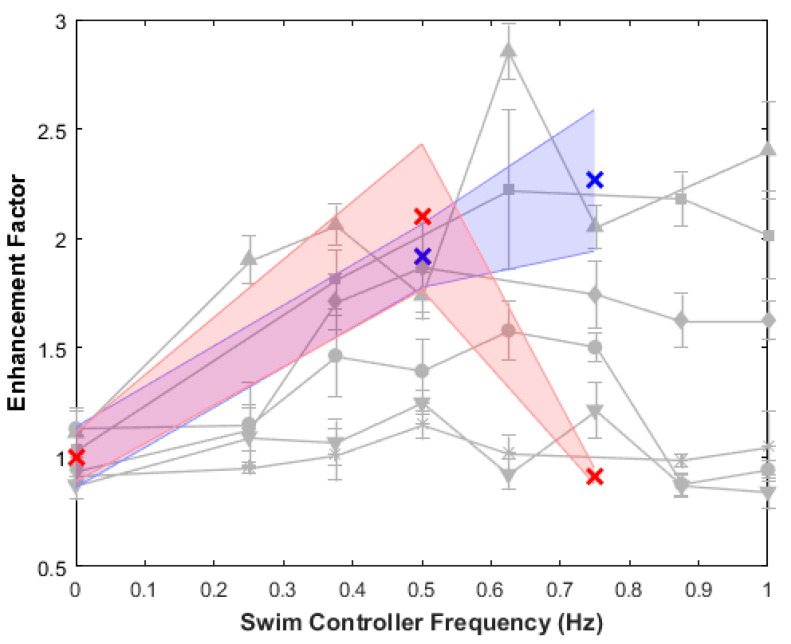
Enhancement factors measured in field work compared to prior work in the lab. The enhancement factor is defined as the swimming speed of each trial divided by the control case at 0 Hz, in which the swim controller is embedded but inactive. An experimentally driven frequency trial for each animal (*n* = 2, shown in blue or red) has been normalized to its own control trial. Prior laboratory work from similar vertical swimming experiments is shown in gray as a comparison, with symbol shape representing each individual animal (*n* = 6) [32]. Variability in the enhancement factor is influenced by the animals’ baseline swimming frequency, in the absence of stimulation.

Swimming speeds generally increased with increasing frequency, although higher frequencies can decrease swimming speeds, as shown by the red data point at an externally driven frequency of 0.75 Hz in Figure 6A. This result confirms previous results of vertical swimming experiments in the laboratory, which showed peak swimming speeds at swim controller frequencies of 0.50 or 0.62 Hz [32]. To compare, the enhancement factors (the swimming speed divided by a baseline swimming speed in which the microelectronic system is embedded but inactive, i.e., the control case at 0 Hz) of both field data and prior work in the laboratory are plotted in Figure 7.

The blue data point at 0.75 Hz shows the highest recorded swimming speed of the dataset, with a linear trend in vertical speed as measured swimming frequency increased. As this biohybrid robotic jellyfish was not tested at higher external frequencies, such as 0.88 or 1.00 Hz, it is unclear whether the maximum enhancement occurred at 0.75 Hz or at which frequency the speed would maximize otherwise. Kinematic analyses of the bell morphology over contraction and relaxation times suggest that a maximum would occur no greater than 1.4 Hz, a proposed biological limit from previous research on muscle refractory periods in scyphozoan physiology [37]. However, at unusually high frequencies driven by the swim controller, such as 1.00 Hz, the bell morphology shifts to a more contracted phase over a longer period of time, in which the muscle ring cannot relax before the subsequent contraction, thereby decreasing the subumbrellar volume to decrease thrust and swimming speeds [32]. This bell morphological change was clearly observed in one animal, externally driven at 1.00 Hz, from the second dive. In that experimental condition, the biohybrid robotic jellyfish had a visibly slower swimming speed and never traversed the entire depth to the ocean surface, as opposed to other trials.

The effects of background flow on animal displacements were also calculated, with an example illustrated in Figure 5B to show oscillatory horizontal displacements resulting from surface currents (with wind speeds of 3.9 m s^−1^, West-Southwest), compared to non-oscillatory vertical displacements (example in Figure 4). As shown in Figure 5A, the main component of animal displacement was in the vertical direction.

In addition to the swimming performance of the overall biohybrid system, the microelectronic components were capable of performing for over 1.5 h when run at 0.50 and 0.75 Hz, and over 45 min when run at 1.00 Hz, entirely submerged and exposed to natural conditions. Furthermore, the microelectronic system stayed embedded in the animals during each set of experiments despite physical handling and flow conditions (for a total of 15 min per system) until user removal for subsequent tests. 

### 3.2. Comparison of Theoretical and Experimental Swimming Speeds 

**Figure 8:**
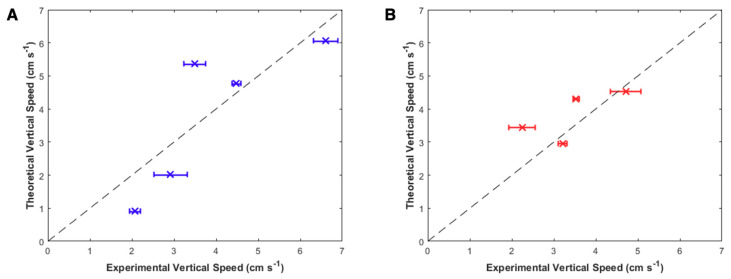
Theoretical versus experimental vertical swimming speeds. Theoretical vertical speeds from the hydrodynamic model versus the measured experimental vertical speeds for each individual jellyfish (**A**) blue using the input parameters delineated in the top row of Table 2 and (**B**) red using the input parameters delineated in the bottom row of Table 2. Inputs were obtained using morphological and time-dependent parameters for each jellyfish. The line of unity is plotted as black dashed lines.

To determine whether theoretical models can predict swimming speeds for future applications to improve user controllability of the system, hydrodynamic models were run using input parameters from the videos of the highest measured swimming speeds for each animal and run at all measured frequencies of that animal. As shown in Figure 8, the theoretical swimming speeds matched the trends in experimental swimming speeds, with mean differences between theoretical and mean experimental vertical speeds of 1.0 ± 0.6 cm s^−1^ and 0.6 ± 0.5 cm s^−1^ for each animal, respectively. In addition to capturing the trends in swimming speeds, the model predicts the variations in swimming performance between the two animals at 0.75 Hz, including greater sensitivity to frequency changes in animal 1, as opposed to decreased sensitivity in animal 2.

## 4. Discussion

The results of this in situ study suggest that biohybrid robotic jellyfish exhibit enhanced swimming modes, even in the presence of real-world conditions. Maximum enhancement factors for the *n* = 2 animals in field experiments were 2.3 ± 0.3 and 2.1 ± 0.3, and absolute swimming speeds increased two- to threefold. Despite limited field data, this corroborates laboratory experiments that reported user control of jellyfish swimming frequencies to enhance swimming speeds over twofold. Furthermore, comparable swimming speed enhancements in the field show a proof of concept that we can predictably improve jellyfish swimming speeds, even with background flows caused by winds of 3.9–4.6 m s^−1^ that resulted in oscillatory horizontal motion (Figure 5), and potential interactions with other animals, such as the fish, ctenophores, and other medusae present in field experiments (Figure 3).

This study also shows the robustness and reliability of the robotic system in these real-world conditions. The electronics for all four animals were viable for at least 45 min to 1.5 h submerged in natural saltwater, dependent on the stimulation frequency. Future studies can conduct field experiments in more locations, including in open water or at greater depths farther from the shore.

To create a more user-controllable biohybrid robotic system, we need to comprehensively study both the natural animal system and how the robotic system interacts with the animal. For example, endogenous swimming frequencies occurred from 0.09 to 0.50 Hz in the absence of external frequency control. However, this range is narrower when we consider each animal separately; the natural pulse response observed in one individual animal ranged from 0.09 to 0.20 Hz, whereas the response in a second animal ranged from 0.40 to 0.50 Hz. Although the present study did not synchronize the externally triggered muscle contractions with the animals’ natural contractions, future studies can examine whether synergistic timing affects propulsion.

The hydrodynamic model we describe can be used to determine which animals are appropriate for optimal robotic integration. By using morphological and time-dependent input parameters from the videos of these animals at only one swimming speed, we predicted the vertical swimming speeds at all frequencies, with a mean error of 0.8 cm s^−1^ (Figure 8). The model captures animal behavior at each of the nine test cases, including predicting doubled enhancements at 0.50 Hz for both animals, as well as the disparity at 0.75 Hz between increased speed in animal 1 and decreased speed in animal 2. Although this jetting model does not incorporate the full hydrodynamics of rowing propulsion evident in *A. aurita*, this simple model is a useful first order prediction. As the results of these experiments validate the hydrodynamic model and trends in swimming speeds, further studies can systematically determine which bell morphological parameters most affect swimming speeds or other metrics of maneuverability through both theoretical modeling and experiments. Regardless, the current model shows utility by predicting the swimming speeds and variations in both animals.

Regarding maneuverability, the current study is limited to purely vertical swimming, ballasted by the swim controller to maintain its upright position. However, future studies can use an unstably balanced weighting system and asymmetric activation of electrodes to allow turning. Accelerometers on both the animal and camera systems can also be used to track 3D motion of the biohybrid robotic jellyfish for more complicated jellyfish maneuvering, such as following trajectories with closed-loop controls. 

The present study also examined horizontal swimming speeds as a proxy for background flow conditions, by taking advantage of coastal conditions to assume primarily horizontal surface currents [38]. These horizonal ocean currents were less likely to affect the vertical swimming speeds exhibited by the biohybrid robotic jellyfish with their ballasted design. Additionally, experimental trials were conducted successively in a narrow span of time to minimize more extreme variations in flow conditions among subsequent trials, with both dives occurring for one hour per day. Future in situ studies can determine how various background flows affect jellyfish swimming using particle image velocimetry, and more laboratory experiments to systematically characterize the user control of jellyfish swimming can also include studies of controlled background flows and their effects on swimming speeds. 

Finally, the main limitation of the current work is the small sample size due to challenges in field work and conditions. Nevertheless, the results demonstrate a proof of concept that a biohybrid robotic jellyfish system can perform at doubled speeds predictably in situ, with the potential for wider use in ocean monitoring after further design modifications. User control of jellyfish swimming has been established for unidirectional swimming in prior and current work. By using the biohybrid robotic jellyfish system in this work as a basis, future experiments can focus on animal maneuverability and robotic design. Suggestions include determining the electrode stimulation patterns needed for asymmetrical swimming and trajectory tracking in the laboratory, adding sensors to collect data from the environment, and integrating biodegradable electronic components for field measurements. 

## 5. Conclusions

The present study demonstrates a proof of concept that biohybrid robotic jellyfish can be implemented in coastal conditions, with doubled swimming speed enhancements, comparable to prior experiments conducted in the laboratory. Differences in the animals’ baseline swimming frequencies could determine sensitivity to robotic manipulation, to address the variability seen in both current and prior work. A theoretical model was developed to predict experimental swimming speeds with mean errors of 0.8 cm s^−1^, using input parameters estimated from videos of one trial to extrapolate speeds at all frequencies for that individual animal. The model accurately predicted variability in swimming speeds among the animals to provide a basis for choosing which animals would be optimal for robotic manipulation in the future. Therefore, this work addresses open questions in the user control of jellyfish swimming, including how real-world environments affect swimming speed enhancements observed in the laboratory, which factors cause large animal variability, and whether theoretical models can predict which individual animals perform better. 

As the biohybrid robotic jellyfish in this study have operated with predictable user control under field conditions, future work can use this existing microelectronic and live animal system in situ as an alternative method to monitor the ocean. By improving maneuverability and incorporating sensors to track environmental changes (such as salinity, acidity, and temperature) into the present design, we can potentially use biohybrid robotic jellyfish as a ubiquitous and energy-efficient tool.

## Figures and Tables

**Table 1:** Field test variables. Animals (*n* = 4) and swim controller frequencies tested in situ. Italicized conditions were tested on the first day and used for quantitative analysis.

Jellyfish	Swim Controller Frequencies (Hz) Tested	Dive Condition Mean Wind Speeds (m s^−1^) and Directions [34]
*1*	*0, 0.50, 0.75*	*3.9 m s^−1^* *West-Southwest*
*2*	*0, 0.50, 0.75*	*3.9 m s^−1^* *West-Southwest*
3	0, 0.50, 1.00	4.6 m s^−1^ West-Southwest
4	0, 0.75	4.6 m s^−1^ West-Southwest

**Table 2:** Input parameters for the hydrodynamic model. Parameters include measured swimming frequency (*f*), relaxed bell diameter (*d_r_*), maximum change in diameter between relaxation and contraction states (Δ*d*), relaxed bell height (*h_r_*), maximum change in height between contraction and relaxation states (Δ*h*), manubrium tissue height (*h_j_*), contraction time (*t_c_*), and relaxation time (*t_r_*).

Animal	*f* (Hz)	*d_r_* (cm)	Δ*d* (cm)	*h_r_* (cm)	Δ*h* (cm)	*h_j_* (cm)	*t_c_* (s)	*t_r_* (s)
1	0.09 0.20 0.47 0.53 0.81	11.3	6.0	4.4	1.0	2.0	0.70	0.73
2	0.40 0.50 0.53 0.75	9.8	5.0	4.2	1.0	2.0	0.87	0.90

## Appendix A

**Table A1:** Experimental parameters and results for field experiments. The diameter, fineness ratio (defined as the ratio of the bell height to diameter), swim controller frequency, measured frequency from counting animal pulses, and calculated vertical swimming speeds for *n* = 2 animals (labeled as blue and red to match plot figures).

Animal	Diameter (cm)	Fineness Ratio	Swim Controller Frequency (Hz)	Measured Frequency (Hz)	Vertical Swimming Speed (cm/s)
1 (blue)	11.3 ± 1.4	0.39	0 (control)	0.09	2.1 ± 0.1
1 (blue)	11.3 ± 1.4	0.39	0.50	0.53	3.5 ± 0.3
1 (blue)	11.3 ± 1.4	0.39	0.50	0.47	4.5 ± 0.1
1 (blue)	11.3 ± 1.4	0.39	0 (control)	0.20	2.9 ± 0.4
1 (blue)	11.3 ± 1.4	0.39	0.75	0.81	6.6 ± 0.3
2 (red)	9.8 ± 0.8	0.43	0 (control)	0.40	2.2 ± 0.3
2 (red)	9.8 ± 0.8	0.43	0.50	0.53	4.7 ± 0.4
2 (red)	9.8 ± 0.8	0.43	0 (control)	0.50	3.5 ± 0.1
2 (red)	9.8 ± 0.8	0.43	0.75	0.75	3.2 ± 0.1

## Appendix B

Although jellyfish are invertebrates that are not under consideration of the Institutional Animal Care and Use Committee (IACUC), the authors would like to address ethical concerns about these animal experiments and incorporating live animals into biohybrid robotic constructs. Jellyfish do not have a centralized nervous system and have no known pain receptors. There is no research that suggests that these animals can feel pain, but regardless, we know that jellyfish secrete excess mucus when stressed. If we use their stress response as a proxy for pain, animals did not exhibit stressed behavior during or after experiments. Furthermore, animals did not show any side effects after the robotic devices were removed; animal behavior returned to its normal state, including normal feeding behaviors. We closely monitored the animals for such stress responses, using the principles of precaution and minimization in accordance with the 3Rs: reduction in animals (smaller sample sizes), replacement with non-animal alternatives (theoretical modeling), and refinement of procedures to minimize potential harm.

Regarding introducing more animals into different ecosystems in the ocean, *A. aurita* are naturally found in Woods Hole. However, because no animals were observed during field experiments, we introduced *A. aurita* and monitored the biohybrid robotic jellyfish carefully to ensure no jellyfish or electronic debris was left in the ocean after experiments. To address these issues in future experiments, further work can include using jellyfish found in their natural habitats, given the ubiquity of *A. aurita*, and incorporating biodegradable electronics already used for medical purposes. Further discussions of the ethical considerations are available in [39].

## Appendix C

Data are available in the Stanford Digital Repository: https://purl.stanford.edu/mh950tt5866.
